# Emergency Laparoscopic Reversal of One‑Anastomosis Gastric Bypass Following Early Anastomotic Failure: Video Case Report of Management

**DOI:** 10.1007/s11695-026-08492-w

**Published:** 2026-02-12

**Authors:** Ata Maden, Ronit Grinbaum

**Affiliations:** 1 Department of General Surgery, Hadassah Medical Center, Jerusalem, Israel; 2 Hebrew University of Jerusalem, Jerusalem, Israel

**Keywords:** One‑anastomosis gastric bypass, OAGB reversal, Anastomotic leak, Bariatric surgery, Emergency surgery

## Abstract

**Background:**

One anastomosis gastric bypass (OAGB) is the third most common metabolic bariatric surgery. Early anastomotic failure is uncommon and may necessitate re-operation. The most common re-operative strategy is peritoneal washout and drainage, while the preferred reconstructive approach is conversion to Roux-en-Y gastric bypass (RYGB). Reversal to normal anatomy in this emergency setting is extremely rare. We present a detailed single-patient emergency OAGB reversal for a leak with operative video, addressing a gap in the published literature.

**Case Presentation:**

A 28‑year‑old woman with BMI 36.2 kg/m² without comorbidities underwent laparoscopic OAGB. Two weeks later, she presented with abdominal pain, nausea, and vomiting. Computerized tomography (CT) demonstrated free air and perianastomotic fluid without contrast extravasation. Laparoscopy revealed anastomotic dehiscence with murky bile-stained fluid largely walled off by the liver. After taking down the anastomosis, a markedly distended gastric remnant prevented RYGB reconstruction. As the patient became unstable, reversal of OAGB was performed as a salvage procedure. Post‑operatively she required intensive care and parenteral nutrition. An intraluminal drain–related leak was diagnosed and managed endoscopically by drain repositioning. She resumed oral intake gradually and was discharged in good condition. At two weeks she tolerated a soft diet. At six months she reported intermittent vomiting with solids, though CT and endoscopy were unremarkable and symptoms improved with dietary modification.

**Conclusion:**

Early anastomotic dehiscence after OAGB may require urgent surgical intervention. When re‑anastomosis or conversion to RYGB is unfeasible and the patient is unstable, reversal to normal anatomy can serve as a salvage option.

**Graphical Abstract:**

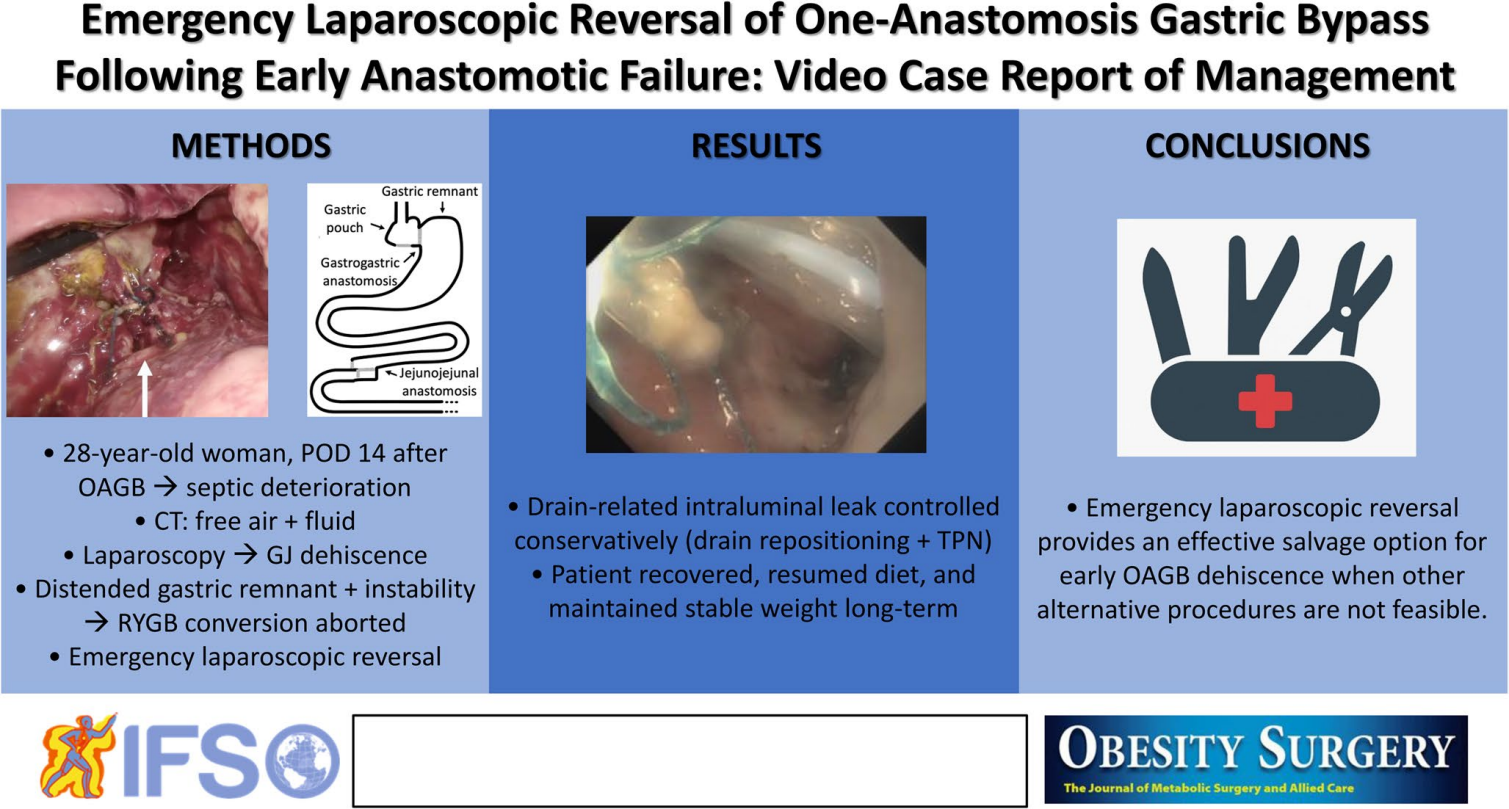

**Supplementary Information:**

The online version contains supplementary material available at 10.1007/s11695-026-08492-w.

## Key Points

Early OAGB leaks are rare; most operated cases undergo lavage or conversion to RYGB.

Emergency reversal is a viable salvage option when other strategies are not feasible.

Emergency OAGB reversal for an acute leak is exceptionally uncommon.

We fill a gap in literature with a detailed single-patient report with operative video.

**Supplementary Information:**

The online version contains supplementary material available at 10.1007/s11695-026-08492-w.

## Introduction

One-anastomosis gastric bypass (OAGB) is among the most commonly performed metabolic bariatric surgeries worldwide, following sleeve gastrectomy and Roux-en-Y gastric bypass (RYGB) as reported in the latest IFSO Global Registry [1]. While the overall proportion of OAGB procedures has shown a modest increase in recent years, its use remains lower than SG and RYGB. OAGB is valued for its relative technical simplicity and comparable metabolic outcomes, yet it carries specific risks including bile reflux, marginal ulceration, nutritional deficiencies and anastomotic leak [2, 3]. In a systematic review of 46 studies involving 44 318 patients, the prevalence of leaks was approximately 1%, with 62.1% requiring surgical re‑intervention [4]. Peritoneal lavage (washout) and drainage was the most common operative approach (30.8%), while conversion to RYGB was the predominant conversion option (9.6%) even though conversional surgery was uncommon overall.

Reversal of OAGB is rare, almost always performed electively for late complications (such as severe protein-energy malnutrition, hypoalbuminemia, excessive weight loss) and only about 1% of patients undergo reversal [5]. While emergency reversal has been scarcely reported in literature, little to no clinical or operative detail is documented [6, 7]. To our knowledge, no single‑patient case report has detailed an emergency reversal performed for anastomotic leak after OAGB.

Here, we describe the diagnosis, operative strategy and post‑operative course of a young woman who developed anastomotic dehiscence two weeks after OAGB. This case demonstrates that emergency laparoscopic reversal can serve as the salvage procedure when other repair and revision/conversion options are not practical.

## Methods

### Patient Information

A 28-year-old female with Body Mass Index (BMI) 36.2 kg/m² (obesity category II) no history of comorbidities, prior surgery, medication use, or smoking underwent elective laparoscopic OAGB at another institution. The early postoperative course was unremarkable and she was discharged on a soft diet with esomeprazole 20 mg twice daily.

Fourteen days after the operation, the patient presented to our emergency department with three days of upper abdominal pain with loss of appetite, and one day of nausea and vomiting. She reported stool passage three days prior to her admission and passed minimal gas since. There was no history of steroid or non-steroidal anti-inflammatory drug use. On admission, vital signs were: temperature 37 °C, heart rate 114 bpm, blood pressure 130/77 mmHg, and respiratory rate within normal limits.

### Clinical Findings and Diagnostic Assessment

**Fig. 1:**
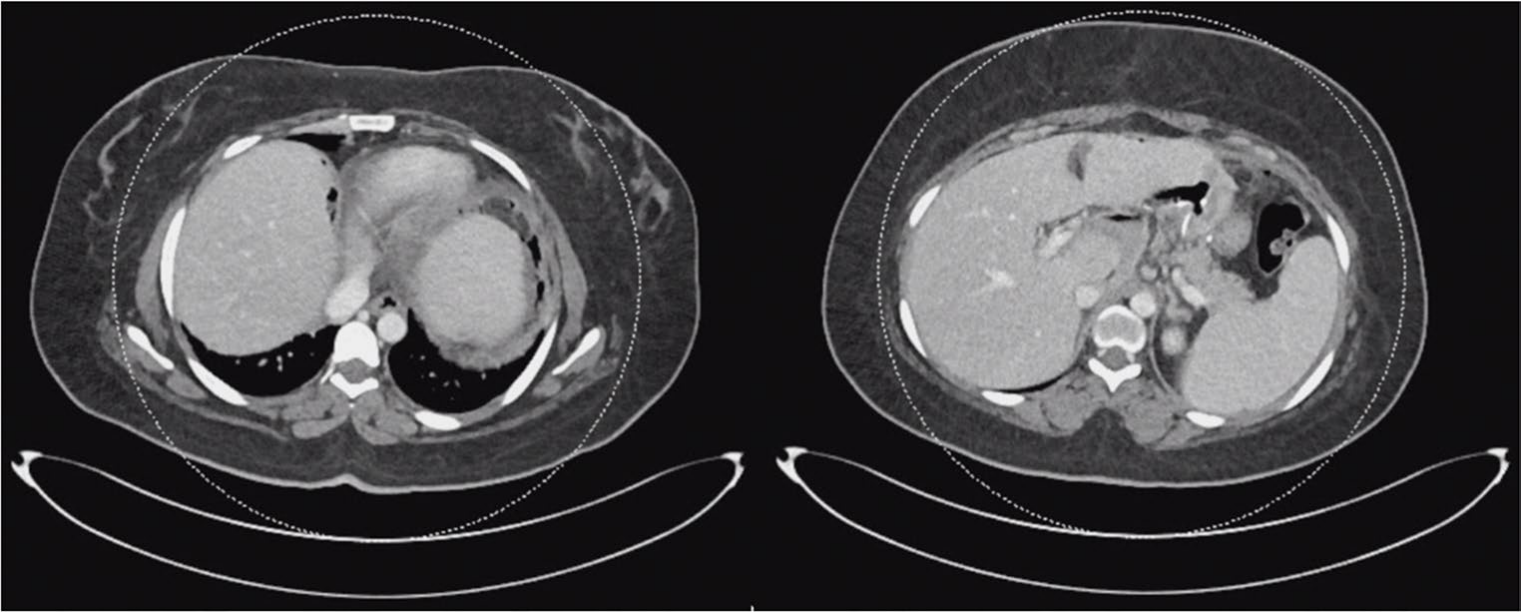
Axial contrast-enhanced computed tomography demonstrating free intraperitoneal air and perianastomotic fluid surrounding the gastrojejunal anastomosis, without contrast extravasation

Physical examination revealed a distended abdomen with tenderness in the upper and left upper quadrants but no signs of peritonitis. Laboratory tests showed leukocytosis (13.5 × 10⁹/L) and elevated C‑reactive protein (31.34 mg/L). Serum lactate, glucose, renal function tests were normal. Electrocardiogram demonstrated normal sinus rhythm; chest radiography showed no pathology. A computed tomography (CT) scan of the abdomen and pelvis with oral contrast revealed free air and fluid around the gastro‑jejunal anastomosis without obvious extravasation or collection (Fig. 1).

The initial differential diagnosis included an anastomotic leak or dehiscence. Management options considered were continuing the conservative therapy that was already started in the emergency department (nil by mouth, broad‑spectrum antibiotics and antifungal therapy), endoscopic evaluation with possible stenting or clipping, and surgical exploration. Given her clinical status and the presence of free air suggestive of early anastomotic leak, exploratory laparoscopy was planned.

Repeat evaluation inside the surgery room showed clinical deterioration, justifying the planned surgery further: temperature 38.4 °C, heart rate 150 bpm and blood pressure 154/65 mmHg. Laboratory values revealed worsening leukocytosis (16.7 × 10⁹/L), increased lactate (3.2 mmol/L), and hypokalemia (2.6 mmol/L).

## Results

### Exploratory Laparoscopy

**Fig. 2:**
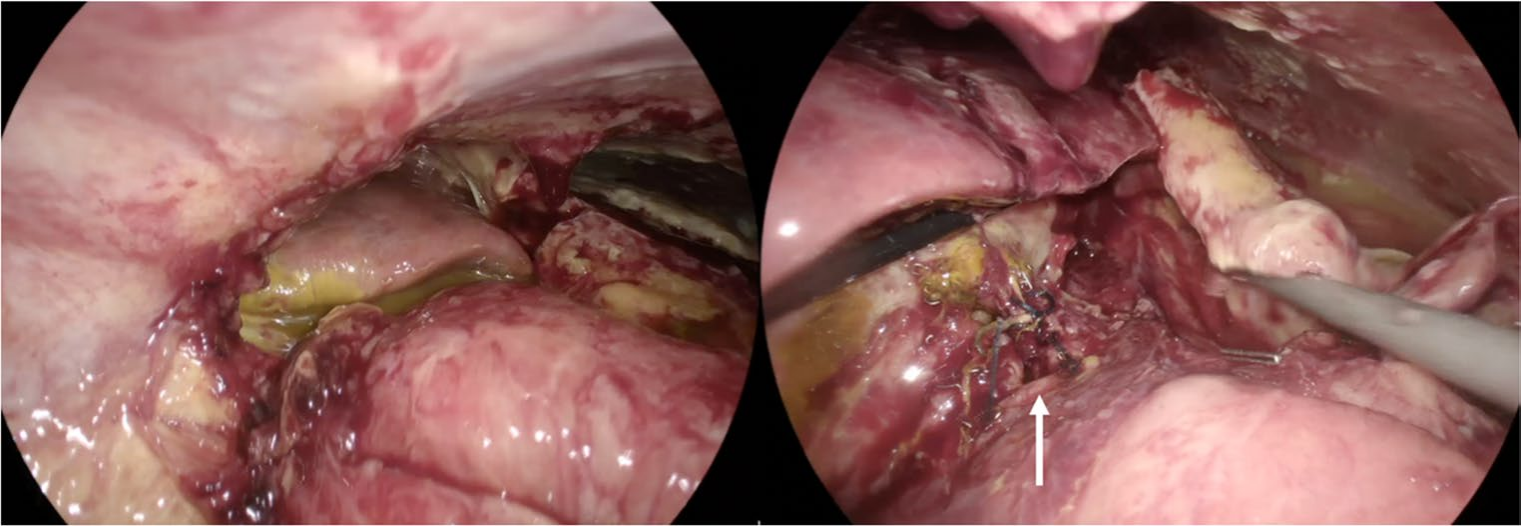
Intraoperative laparoscopic views demonstrating early anastomotic failure. Left: Murky, bile-stained fluid with associated inflammatory changes. Right: Completely dehisced gastrojejunal anastomosis (arrow)

An experienced laparoscopic bariatric team performed the procedure. Under general anesthesia, the abdomen was accessed via a 12-mm optical trocar in the supra-umbilical position. During the procedure, four additional trocars were placed in the upper abdomen. Laparoscopic exploration, after brief adhesiolysis, revealed dehiscence of the gastro‑jejunal anastomosis with murky, bile‑stained fluid and inflammatory changes in the upper abdomen. The main collection was walled off by the liver, and there was no evidence of generalized peritonitis (Fig. 2).

**Fig. 3:**
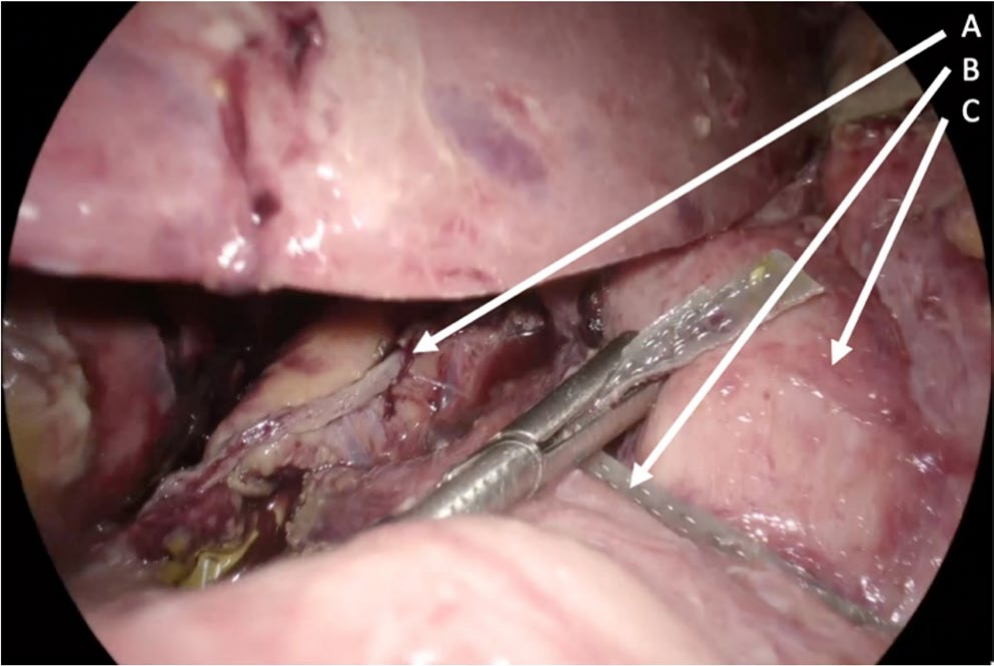
Intraoperative laparoscopic views showing inability to advance the efferent limb to the newly created gastric pouch. Arrow A: gastric pouch; Arrow B: efferent limb; Arrow C: gastric remnant

After dissection, the gastric pouch, and the anastomosed jejunal loop were identified and the small bowel was run in its entirety. The biliopancreatic (afferent) limb was measured at 110 cm from the ligament of Treitz to the gastrojejunal anastomosis. The OAGB anastomosis was taken down by dividing the gastric pouch just proximal to the gastrojejunostomy and transecting the jejunal limbs both proximal and distal to the anastomosis using 60-mm purple Endo GIA Tri-Staple cartridges. The intervening jejunal mesentery was divided to allow safe mobilization and separation of the limbs. During attempts to construct a new anastomosis, the distended gastric remnant prevented advancement of the efferent limb to the newly formed gastric pouch (Fig. 3).

**Fig. 4:**
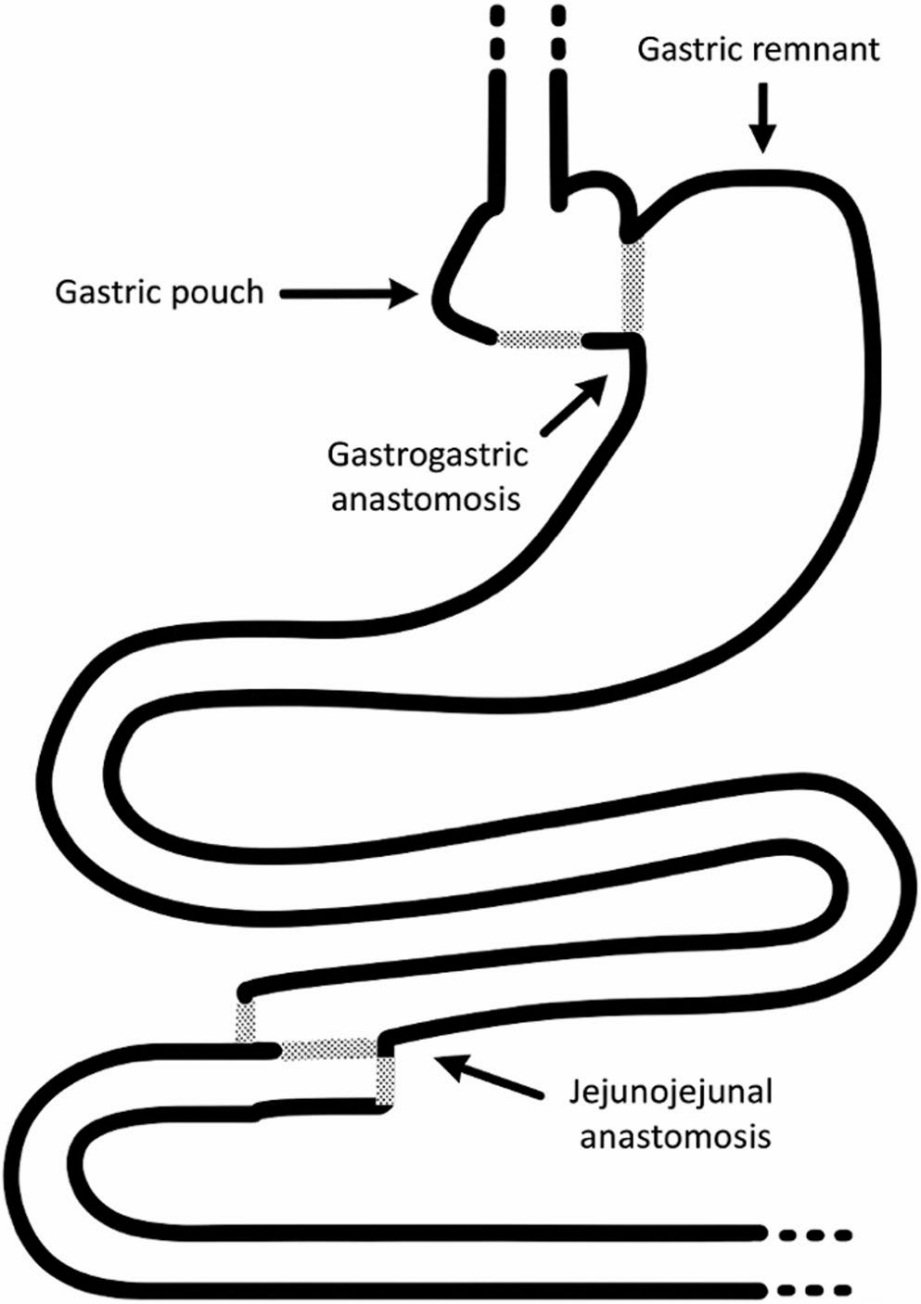
Schematic illustration of the anatomy after completion of the reversal

The patient was developing worsened acidosis and was requiring increased vasopressor support, contraindicating a prolonged reconstruction. As a salvage procedure, it was decided to reverse the OAGB. Resection of the distended remnant with subsequent RYGB reconstruction was also considered but was deemed unsafe due to the patient’s hemodynamic deterioration and the extensive inflammation at the surgical site. The gastric pouch and remnant stomach were approximated with 60-mm purple Endo GIA Tri-Staple cartridge to create a stapled gastrogastrostomy, and the gastrogastrostomy opening was stitched using a continuous barbed suture (V‑Loc). The jejunal limbs were also re‑anastomosed with the 45-mm tan Endo GIA Tri-Staple cartridges and the reversal was completed anatomically (Fig. 4).

Three drains were placed (two in the upper abdomen near the anastomosis and one in the lower abdomen). The patient received intra‑operative potassium chloride, packed red blood cells and fresh frozen plasma and was transferred intubated and on pressors to the intensive‑care unit.

### Post‑operative Management

The patient remained on total parenteral nutrition, broad‑spectrum antibiotics (covering the cultures taken during the operation that were positive for Streptococcus anginosus, Clostridium perfringens and Escherichia coli) and antifungal therapy, with low-molecular-weight heparin for deep‑vein thrombosis prophylaxis. Thiamine supplementation was administered prophylactically due to prolonged NPO status and increased risk of deficiency after bypass surgery. She was weaned from vasopressors by post‑operative day (POD) 2 and extubated on POD 3. Sequential laboratory tests showed improving inflammatory markers.

**Fig. 5:**
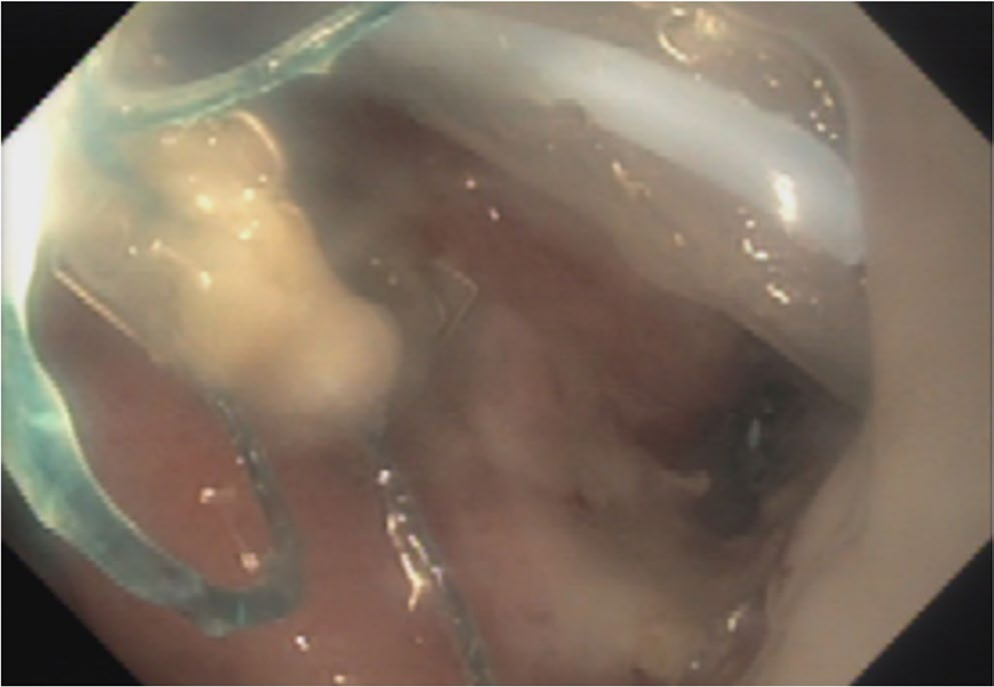
Endoscopic view of the intraluminal drain

On POD 9 one of the upper abdominal drains began to return gastric content, without any change in the clinical status of the patient. Gastroscopy, performed by a gastroenterologist with the surgeon present in the room, revealed a small luminal opening at the anastomosis site, with the drain protruding intraluminally. Under endoscopic guidance, the surgeon withdrew and re-advanced the drain into the intra-abdominal cavity. A nasogastric tube was placed and the patient remained nil by mouth on total parenteral nutrition (Fig. 5).

Subsequent CT scanning on POD 15 showed no collection or leak. The nasogastric tube was removed on POD 16, and oral intake was advanced gradually to a soft diet under dietitian supervision. Total parenteral nutrition, antibiotics and antifungal agents were discontinued. A barium swallow study on POD 19 demonstrated normal passage of contrast without leakage. She was discharged home on POD 22 on esomeprazole 20 mg twice daily with low‑molecular‑weight heparin and advised to continue a soft diet.

### Clinical Outpatient Follow‑up

**Fig. 6:**
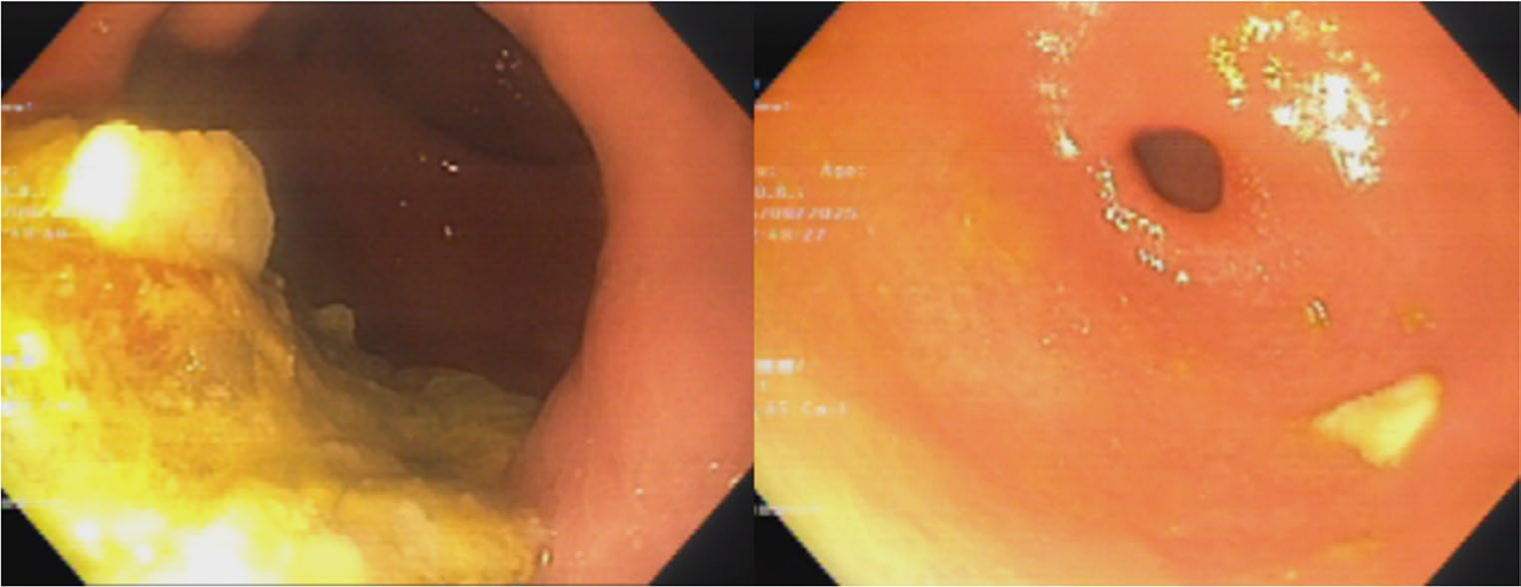
Endoscopic images showing retained intragastric contents in the remnant (left) and a patent outflow tract with normal duodenal passage (right)

Good tolerance of a soft diet was reported on two‑week follow‑up at outpatient clinic, with normal bowel movements. Esomeprazole was tapered to 20 mg once daily. At one-month, clinical follow-up with the institution dietitian showed good tolerance to solid food that was introduced. At six‑month follow‑up she maintained a normal diet and had normal bowel function. Occasional early satiety with rare, intermittent vomiting with solid food was reported. CT with contrast and gastroscopy were unremarkable other than some retained intragastric content at the anastomosed remnant (Fig. 6).

Dietary modifications with dietitian follow-up alleviated her symptoms, and subsequently she maintained adequate nutritional status.

### Longitudinal Weight Trend

Before the initial OAGB surgery the patient weighed 86 kg (BMI 36.2 kg/m²). After the reversal surgery and prolonged hospitalization, she was discharged at 75.5 kg (BMI 31.8 kg/m²). At 1-month follow-up her weight had decreased further to 72.4 kg (BMI 30.4 kg/m²), and by 6 months she had stabilized around 76 kg (BMI 32 kg/m²).

## Discussion

Early anastomotic failure after OAGB is a rare but life-threatening complication that poses complex challenges in clinical decision-making. In the presence of diffuse contamination or instability, surgical re-intervention is often unavoidable. Current evidence and expert opinion strongly favor early surgical source control in such scenarios [8, 9].

According to the IFSO Worldwide OAGB Survey, RYGB represents the predominant conversion approach when treating leaks after OAGB [10]. Published literature supports urgent conversion to RYGB as an effective strategy for diverting bile away from the leak and achieving sepsis control [4, 11].

As previously noted, reversal is well documented for late sequelae, but for acute leaks it is reported only anecdotally and without procedural detail [5–7].

In our patient, the decision to reverse the OAGB was driven by the intraoperative conditions and the patient’s physiologic deterioration. A dehisced gastrojejunostomy with contamination and septic shock required rapid source control. After takedown of the anastomosis with the intention of performing RYGB, the markedly distended gastric remnant prevented safe advancement of the efferent limb to the gastric pouch. At this point, resection of the gastric remnant and proceeding with RYGB was also considered, but the patient’s instability and the hostile surgical site made this option likely to prolong or complicate the operation. With the gastric remnant lying immediately adjacent to the pouch, laparoscopic reversal to normal anatomy was selected as the safest and the fastest option.

Notably, minimally invasive completion of the reversal was achieved. Although some surgeons might resort to an open laparotomy in such septic conditions, multiple studies indicate that, in select patients, early postoperative leaks after bariatric surgery can be managed laparoscopically by experienced teams, with acceptable outcomes [12–14].

The postoperative course highlighted the complexity of recovery after emergency reversal. The patient developed a drain-related intraluminal communication. Such a complication is not unexpected; even in elective reversal series, anastomotic leaks have been observed [15]. Our management prioritized conservative therapy: the surgical drain was repositioned back into the abdomen and the treatment with broad-spectrum antibiotics with nasogastric tube and TPN was continued. The strategy was effective and the leak gradually resolved. This aligns with prior studies on post-bariatric leaks that once adequate drainage is established and sepsis is controlled, supportive care can lead to leak closure over time [6, 16].

Finally, this case highlights that reversal to normal anatomy is a legitimate and important addition to the surgeon’s armamentarium in managing OAGB anastomotic failures. The trade-off, of course, is loss of the bariatric effect, which can be a necessary sacrifice when patient stability and sepsis control take precedence. In our patient, the weight loss initially plateaued, and at subsequent follow-up there was a slight weight regain.

This case is, to our knowledge, the first detailed report of an emergency OAGB reversal for an acute anastomotic dehiscence. It provides operative and clinical insights that may guide surgical teams faced with a similarly challenging situation. As global OAGB volumes rise, rare complications like this may be encountered more frequently. Ultimately, recognizing when to abandon the standard algorithm and perform a reversal can be life-saving, and this option should be kept on the table when other solutions fail or the patient’s condition dictates a salvage approach.

## Conclusion

Early anastomotic failure after OAGB is rare and often requires urgent operative management. Although washout, drainage, and conversion to RYGB are the most commonly performed surgical approaches for early leaks, anatomical limitations or physiologic instability may render these strategies unfeasible. In such situations, reversal to normal anatomy can provide a decisive salvage solution. This report provides a detailed description of an emergency laparoscopic reversal for early OAGB dehiscence. It gives operative and clinical insight, demonstrating that timely reversal by an experienced team can achieve a favorable outcome.

## Supplementary Information

Below is the link to the electronic supplementary material.

Supplementary Material 1

## Data Availability

No datasets were generated or analysed during the current study.
